# Cost-Utility Analysis of Treatments for Early Childhood Caries in Remote Aboriginal Communities

**DOI:** 10.1177/23800844251346743

**Published:** 2025-08-16

**Authors:** U. Tonmukayakul, S. Kularatna, D. Atkinson, L. Jamieson, P. Arrow

**Affiliations:** 1 Deakin Health Economics, Institute for Health Transformations, Deakin University, Geelong, VIC, Australia; 2 Australian Centre for Health Services Innovation, School of Public Health and Social Work, Queensland University of Technology, Brisbane, QLD, Australia; 3 Health Services and System Research, Duke-NUS Medical School, Singapore; 4 Rural Clinical School of Western Australia, University of Western Australia, Broome, Western Australia, Australia; 5 Australian Research Centre for Population Oral Health, Adelaide Dental School, The University of Adelaide, Adelaide, SA, Australia; 6 Western Australia Dental Health Services, Perth, WA, Australia; 7 Dental School, University of Western Australia, Perth, WA, Australia

**Keywords:** dental caries, atraumatic restorative treatment, Hall technique, economic evaluation, Indigenous health, quality-adjusted life-years

## Abstract

**Introduction:**

Early childhood caries is a significant problem affecting Aboriginal preschoolers in remote communities who lack access to dental services. A trial was conducted to assess the benefits of atraumatic restorative treatment combined with the Hall technique (ART-HT) versus usual care for managing early childhood caries in this population.

**Objectives:**

This trial-based cost-utility analysis evaluates costs and quality-adjusted life-years (QALYs) of ART-HT compared with usual care within a 1-y time frame.

**Methods:**

A decision-analytic model simulated the costs and QALYs for 2 scenarios: trial service delivery and minimum ART-HT delivery. The incremental cost per QALY gain ratio (ICER) was calculated for each scenario. QALY were estimated using the Early Childhood Oral Health Impact Scale-4D (ECOHIS-4D) and adjusted for baseline. Probabilistic sensitivity analyses were conducted to assess the robustness of the base-case results. Cost-effectiveness acceptability curves were generated to determine the likelihood of ART-HT being cost-effective at various willingness-to-pay thresholds. All costs are presented in Australian dollars for 2021.

**Results:**

Children receiving ART-HT had slightly higher QALYs than those receiving usual care did (0.85 vs. 0.83). The base-case analysis showed an average ICER of $2,013/QALY gained (95% uncertainty interval −$45,246 to $21,676). In the sensitivity analysis, the average ICER was $2,573/QALY gained (95% uncertainty interval −$43,658 to $23,352). ART-HT had an 85% and 81% chance of being cost-effective at a $15,000/QALY gained threshold in the base-case and alternative scenario, respectively.

**Conclusion:**

ART-HT showed potential cost-effectiveness compared with usual care within the observed period. While QALY gains were modest, ART-HT may offer a valuable strategy to improve service access for Aboriginal children in remote communities.

**Knowledge Transfer Statement:**

This study demonstrates that atraumatic restorative treatment combined with the Hall technique (ART-HT) is cost-effective in managing early childhood caries in remote Aboriginal communities. By providing a viable dental care strategy, ART-HT can improve access to essential dental services, enhance oral health outcomes for Aboriginal preschoolers, and offer value for money, thereby contributing to overall better health in these underserved populations.

## Introduction

Dental caries is a significant public health problem among Aboriginal and Torres Strait Islander (hereafter, Indigenous) children in Australia. According to the Australian Research Centre for Population Oral Health, the rates of dental caries among Indigenous children were 1.5 to 2.5 times higher than the national average (National Health and Medical Research Council and Australian Research Centre for Population Oral Health 2021). Indigenous children aged 0 to 4 y had a 1.6 times higher rate of hospitalization for dental conditions than non-Indigenous children did (Australian Institute of Health and Welfare & National Indigenous Australians Agency 2023). Furthermore, most cases of dental decay in Aboriginal children remain untreated (Piggott et al. 2019).

A report from Western Australia found that Indigenous children aged 2 to 4 y had more extensive dental decay, pain, and severe early childhood caries than their non-Indigenous counterparts did (Dogar et al. 2011). Only 30% of Indigenous children were caries free compared with 75% of non-Indigenous children (Dogar et al. 2011). These findings are consistent with those of previous studies (Smith et al. 2015; Kroon et al. 2019), which also confirmed that untreated decay is a significant issue in Indigenous children.

Improving access is crucial to enhance the oral health of Indigenous children. According to the National Aboriginal and Torres Strait Islander Health Survey, only 58% of Indigenous children aged 0 to 14 y have seen an oral health provider in the past 12 mo (Australian Institute of Health and Welfare 2023b). Moreover, approximately 19% of Indigenous Australians reported that they did not see a dentist when they needed it in the previous 12 mo due to cost (Australian Institute of Health and Welfare 2023b).

Atraumatic restorative treatment (ART) and the Hall technique (HT) have been proposed as alternative approaches for managing early childhood caries, particularly in locations where access to specialized dental care is severely limited (Arrow, Piggott, et al. 2021; Piggott et al. 2021). ART involves the removal of carious lesions using hand instruments without the need for local anesthesia or dental drills. HT is a minimally invasive restoration of primary teeth that involves seating and cementing a preformed metal crown on a decayed tooth, without any tooth preparation, caries removal, or local anesthesia. Both ART and HT can be easily performed in remote communities by dental health professionals, with minimal training and equipment. ART has a high success rate in managing dental caries and has been shown to result in high levels of patient satisfaction (Arrow, Forrest, et al. 2021). One study found that ART required lower costs than dental treatment under general anesthesia did in children living in metropolitan/urban Western Australia (Tonmukayakul et al. 2021).

The combination of ART and HT (ART-HT) has proven effective in managing early childhood caries in Indigenous children residing in rural and remote communities in the Kimberley region of Western Australia (Arrow, Piggott, et al. 2021; Piggott et al. 2021). In addition to the clinical benefits of ART-HT, a recently published cost-effectiveness analysis study found that ART-HT effectively managed dental caries in Indigenous children, providing better oral health outcomes compared with usual care, albeit with modest additional costs (Tonmukayakul et al. 2025). This current economic evaluation focuses on quality-of-life outcomes measured by a novel instrument called the Early Childhood Oral Health Impact Scale-4D (ECOHIS-4D) (Hettiarachchi, Arrow, et al. 2022; Kularatna et al. 2022), using the same dataset from a pragmatic trial as the published cost-effectiveness analysis study. This study aimed to compare the costs and quality-adjusted life-years (QALYs) of ART-HT to usual care to provide distinct information that can help decision makers determine whether ART-HT offers good value for money against a willingness-to-pay threshold of $50,000/QALY. The findings of this economic evaluation can inform policy decisions and resource allocation for the management of dental caries in young children with limited access to dental services.

## Materials and Methods

The culturally appropriate ART-HT trial was a cluster-randomized, assessor-blinded trial that used a parallel stepped-wedge design (Arrow et al. 2018). Eligible participants were Indigenous children younger than 6 y who were recruited from participating communities in the Kimberley region of Western Australia. Children with medical or developmental conditions that could not be treated in a primary care setting or who had acute dental infections that required urgent care were excluded from the study. Signed informed consent was obtained from the parents or guardians of the young participants.

The Kimberley region in northwestern Australia covers approximately 42,000 km^2^ with a population density of 0.09 persons/km^2^. It is a predominantly remote region (97% classified as very remote). Dental care access is constrained by limited service providers, geographical dispersion, the need for 4-wheel-drive vehicles on unsealed roads, and road impassability due to flooding during the 6-mo wet season.

The trial was registered with the ANZ Clinical Trials Registry (ACTRN126001537448) and approved by the Western Australian Country Health Service Human Research Ethics Committee (WACHS HREC Project References 2017/01), the Western Australia Aboriginal Health Ethics Committee (Project Reference: 790), the University of Adelaide Health Research Ethics Committees (H-2017-015), and Deakin Human Research Ethics Committees (2022-084).

The following sections provide a brief overview of the ART-HT trial. The full details of the trial design, selection criteria, amendments, randomization and allocation, assessments, and health economic analysis plan have been reported elsewhere (Arrow et al. 2018; Arrow, Piggott, et al. 2021). This current study was reported in accordance with the CHEERS checklist.

### ART-HT Intervention

School dental therapists who had completed the ART-HT training provided ART-HT procedures to the participants in the intervention group. Minimally invasive ART procedures were performed at local dental clinics, school libraries, or family day care centers using mobile equipment after oral assessments by calibrated examiners at baseline and 1-y follow-up. A radiograph of a tooth was obtained prior to the placement of an HT crown and in all instances of pulp therapy and tooth extraction. ART was always performed as part of the intervention, while HT was used in cases in which ART alone was deemed insufficient for addressing the dental needs of the child, such as in instances of extensive carious lesions. The participating children and their parents were given instructions on toothbrushing and provided with fluoride toothpaste and toothbrushes.

### Usual Dental Services

After the baseline oral health assessment, the children in the control group were informed about their oral health status and advised to obtain care from their usual providers, who may provide services free of charge. The usual sources of care included visiting government dental services (normally 3 times a year), volunteer-based nongovernment dental services (annually), or Royal Flying Doctor Dental Services (at least once a year). Children could also travel to established dental clinics (government or private practice) in larger towns located hundreds of kilometers from their communities.

At follow-up, ART-HT intervention was also offered to the usual care group. However, rigid COVID-19 restrictions have prevented the assessment of delayed ART-HT and visits from usual care providers. Consequently, usual care effectively refers to limited dental services.

### Outcome Measures

This cost-utility analysis focused on QALYs, an outcome measure recommended by national reimbursement authorities such as the Australian Pharmaceutical Advisory Committee (Department of Health and Aged Care 2016) and the UK National Institute for Health and Care Excellence (NICE 2022). QALYs were estimated by multiplying utility values truncated between 0 (death) and 1 (full health) by duration.

The utilities were derived from the ECOHIS-4D Australian value sets (Hettiarachchi, Arrow, et al. 2022). ECOHIS-4D is a parent-completed oral health–related quality-of-life measure for preschool children. The ECOHIS-4D instrument was recently developed based on the ECOHIS questionnaire (Kularatna et al. 2022). The 4 dimensions of the ECOHIS-4D ask parents if their child experienced pain, difficulty eating, irritability, or difficulty talking. Each dimension had 3 response ratings: “hardly ever,” “occasional,” and “never.” The ECOHIS-4D has been validated as a reliable measure of oral health–related quality of life in young children. It effectively distinguishes clinical severity levels and outperforms a generic measure, the Child Health Utility Index (CHU-9D) in correlating with oral health indicators such as dmft (Weerasuriya et al. 2024). An Australian algorithm (Hettiarachchi, Arrow, et al. 2022) was used to estimate the utility weights from the answers to the ECOHIS-4D. ECOHIS-4D data were collected at baseline and follow-up.

The EQ-5D-Y was also administered in the trial. Although the EQ-5D-Y can provide utilities for QALY calculations, Australian value sets are not available. EQ-5D-Y is a generic instrument that is less sensitive to dental caries than the ECOHIS is. Therefore, this study used only utilities from ECOHIS-4D to provide context-specific research findings.

This cost-utility analysis study did not focus on treatment success for failure, nor did it assess the extent of emergency care needed.

### Costs

Two cost components from the health care perspective were considered: ART-HT training and treatment costs. The average ART-HT training was $9.24 per child. In the base-case scenario, ART-HT treatment costs were estimated based on the actual treatment provided by the clinicians of the research team. In the trial, some children did not undergo oral examinations and/or treatment because they were not in their communities at the time. In the hypothetical scenario, ART-HT treatment costs were based on the conservative assumption that every child in the ART-HT group received oral health examinations, education, and fluoride application. This assumption, agreed by research clinicians, was intended to present a more optimistic view of potential treatment options available for children with a high prevalence of dental caries who resided in locations with limited resources.

For the ART-HT group, treatment item codes provided by the clinicians of the research team were recorded and converted into Australian dollars using the 2021 Department of Veterans Affairs Fee Schedule for dentists and dental specialists (Department of Veterans Affairs 2021). Proxy treatment costs were used for the usual care group because information about the dental treatment they received from their usual providers was not available. The proxy costs were based on the average dental treatment costs per child provided by the Western Australian Dental Health Services in the Kimberley region in 2018–2019 and 2019–2020, inflated to 2021 prices. While these proxy costs may not fully reflect the specific circumstances of the Aboriginal population, this was the most reliable information available to us, given the challenges in obtaining data directly from usual service providers in the care group.

Per diem allowances and travel costs were excluded from the analysis. This is because providers for both the ART-HT intervention and usual care, including the Royal Flying Doctor Service, fundamentally use road transport to travel to and from the same communities to provide dental care. We acknowledge that variations in specific route or vehicle types may exist. However, given this similarity in transport mode, we expected travel costs to be broadly comparable in terms of distance and frequency, with any potential differences likely having a minimal net impact.

### Utility Values Adjustment and Comparison

Baseline utilities at follow-up are likely to exhibit a strong correlation with utilities at baseline follow-up, which can significantly affect the calculation of QALY (Manca et al. 2004). QALYs were derived from the area under the curve, incorporating utilities at both baseline and follow-up as well as the duration between the 2 assessments. It is evident that the incremental cost-effectiveness ratio (ICER) is sensitive to minimal changes in its denominator, which represents the QALYs (Manca et al. 2004). Consequently, any imbalance in the mean baseline utilities between the 2 groups can result in an inaccurate ICER, potentially leading to misleading conclusions (Manca et al. 2004).

To avoid misleading results, the follow-up utilities were adjusted for the baseline utility weight of each individual using a regression technique. This adjustment accounts for individuals’ differences and provides a more accurate representation of changes in QALYs attributable to the intervention. STATA Version 17 (StataCorp 2021) was used to perform regression-based adjustment. Differences within and between groups were tested using Wilcoxon matched-pair rank-sum analysis, with a significance level of 0.05.

### Cost-Utility Analysis

Costs and outcomes associated with ART-HT and usual care were constructed using Microsoft Excel. Incremental costs and effects were computed by comparing ART-HT with usual care. The ratios of net cost to net outcome, known as the ICERs, were calculated.

Probabilistic sensitivity analysis was also performed to assess the robustness of the results from the base-case and sensitivity analyses. Excel-based Monte Carlo simulations were conducted by randomly selecting 1 value for each parameter from its range before estimating a new ICER/QALY gain. A new ICER was recorded, and the simulation process was repeated 1,000 times to generate 1,000 possible ICERs. These ICERs were plotted on a 2-dimensional diagram called cost-effectiveness planes, representing the differences in costs on the *y*-axis and QALYs on the *x*-axis.

An intention-to-treat analysis was used in both deterministic and probabilistic analyses, which included all randomized participants, accounting for those lost to follow-up due to being away from their communities during assessment visits. Movement from communities is part of the Aboriginal culture. The main outcome publication arising from this trial found no significant differences in caries experience between followed-up and lost to follow-up participants (Arrow, Forrest, et al. 2021). This suggests that the results from the intention-to-treat analysis would offer a more conservative estimate of treatment effects and reflects real-world scenarios in which adherence may vary.

In addition to the cost-effectiveness plane, cost-effectiveness acceptability curves were created to present the probability that ART-HT is cost-effective within a given range of willingness-to-pay thresholds.

Discounting was not applied to costs and outcomes because the data were collected within a 1-y time frame. The trial successfully gathered baseline and 1-y follow-up data in 2018–2019. However, strict COVID-19 lockdowns subsequently prevented the research team from accessing the participating communities for a 2-y follow-up, including the delayed implementation of ART-HT in the usual care group. This limitation primarily influenced the 1-y time horizon. In addition, there is limited evidence on caries progression and the longevity of dental caries treatments in Aboriginal children. As outlined in the introduction, these children experience a distinct pattern of disease due to a complex interplay of factors including high prevalence of untreated caries, limited access to care, and socioeconomic variables. Extrapolating beyond the trial period using data from non-Aboriginal populations or relying on “best guess” estimates was deemed inappropriate due to the potential for introducing significant bias and generating unreliable projections. Consequently, projections of the costs and effects beyond the trial period were not attempted. The appendix lists the model parameters used in both the deterministic and probabilistic analyses.

## Results

Data from a total of 337 participants (177 ART-HT from 12 communities, 160 usual care from 13 communities) were analyzed. The study parameters can be found in the appendix. The participants in both groups remained in the trial for approximately 11 mo (SD, 0.05 mo). At baseline, the mean utilities for both groups were comparable (0.907 for ART-HT vs. 0.923 for usual care). At follow-up, a small increase in utility was observed in both the groups. The ART-HT group had significantly increased utilities of up to 0.037 out of a full scale of 1 (*P* = 0.01), whereas the usual care group had a non–statistically significant increased utility of 0.012 (*P* = 0.34). The improvement in utilities of the ART-HT group was significantly greater than that of the usual care group (*P* < 0.01). The Table presents the participants’ demographic and utility values.

**Table.** Participant characteristics.

	ART-HT		Usual Care		*P* Value of Baseline Difference between Groups
	Baseline (*n* = 177)	Follow-up (*n* = 122)	Baseline (*n* = 160)	Follow-up (*n* = 106)	
Age, y	3.8 (1.7)		3.3 (1.7)		0.007
Male, *n* (%)	88 (50%)		74 (46%)		0.57
dmfs	9.5 (10.8)	9.9 (10.1)	6.6 (10.6)	7.1 (11.0)	0.001
dmft	4.4 (4.0)	4.6 (3.8)	3.1 (3.8)	3.3 (3.9)	0.001
Total ECOHIS	4.4 (7.5)	4.6 (7.5)	4.0 (6.3)	3.5 (6.0)	0.17*
Observed ECOHIS-4D	0.907 (0.145)	0.944 (0.104)	0.923 (0.117)	0.935 (0.112)	0.159*
Adjusted ECOHIS-4D	0.907 (0.145)	0.944 (0.039)	0.923 (0.117)	0.935 (0.031)	<0.001*

ART-HT, atraumatic restorative treatment combined with the Hall technique; ECHOHIS-4D, Early Childhood Oral Health Impact Scale-4D.

Data are presented as the mean (standard deviation) or *n* (%).

* Statistically significant.

### Findings from the Base-Case Scenario

**Figure 1.**
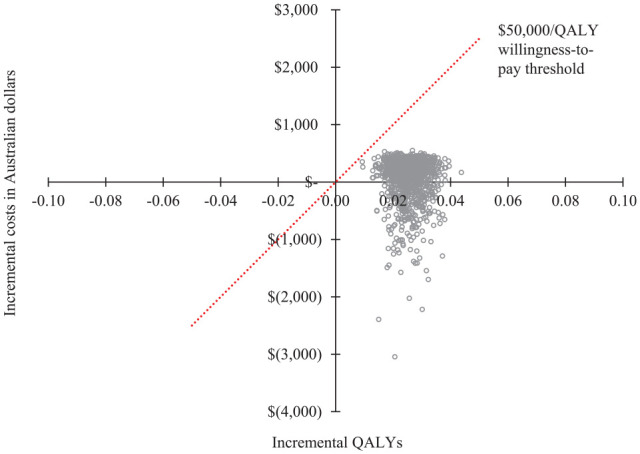
Cost-effectiveness plane of the base-case analysis.

**Figure 2.**
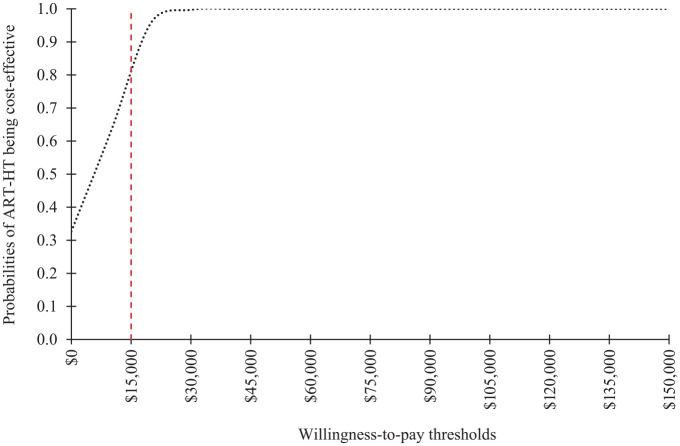
Acceptability curve of the base-case analysis.

In the base-case scenario (the actual ART-HT provision), ART-HT was costlier (mean $46, with the uncertainty interval ranging from −$1,038 to $456) and associated with greater QALY gain (0.025, with the uncertainty interval ranging from 0.016 to 0.036) than usual care was. The average ICER/QALY was $2,013 (uncertainty interval ranging from −$45,246 to $21,676). Figure 1 shows the cost-effectiveness plane with ART-HT being more effective and potentially resulting in cost savings of 37% of simulated ICERs. ART-HT was a cost-effective intervention based on the $50,000/QALY threshold and was highly likely to be cost-effective when the threshold was $15,000/QALY (Fig. 2).

### Findings from a Hypothetical Scenario

**Figure 3.**
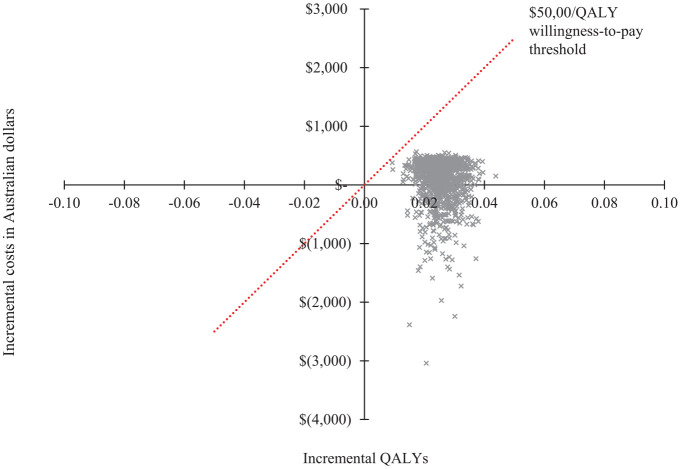
Cost-effectiveness plane of the hypothetical scenario.

**Figure 4.**
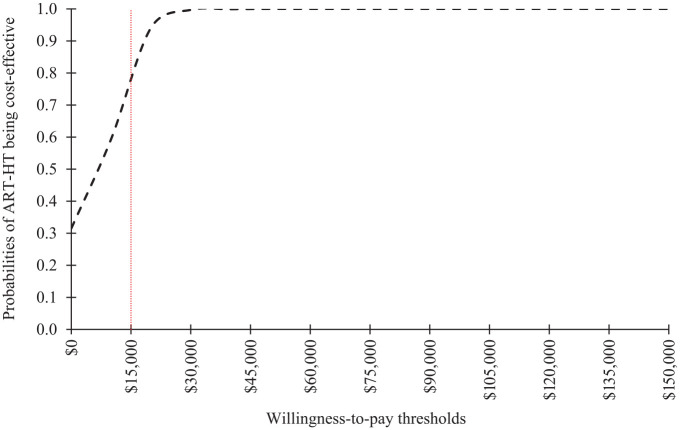
Acceptability curve of the hypothetical scenario.

If the minimum ART-HT procedures were provided, similar findings to those of the base-case analysis were observed, with an average ICER/QALY of $2,573 (uncertainty interval ranging from −$43,658 to $23,352). Approximately 32% of the ICERs were located in the southeast quadrant of the cost-effectiveness plane (Fig. 3), indicating that ART-HT saved costs, coupled with more QALY gain. ART-HT remained cost-effective when compared with the $50,000/QALY threshold (Fig. 3). The probability of ART-HT being cost-effective was high, even when the willingness-to-pay threshold was $15,000/QALY (Fig. 4).

## Discussion

This trial-based economic evaluation demonstrated that the ART-HT approach may offer potential improvements in QALYs and possible cost savings compared to usual care within the 1-y time frame of this study. The findings of this cost-utility analysis contribute to the growing body of literature examining the health and economic benefits of ART-HT in young populations (Tonmukayakul and Arrow 2017; Schwendicke et al. 2019; Arrow and Forrest 2020; Arrow, Piggott, et al. 2021; Tonmukayakul et al. 2021; Arrow et al. 2022; Tonmukayakul et al. 2025).

Within the limitations of this short-term analysis, the results of the base-case and hypothetical scenarios indicated that ART-HT could be a cost-effective alternative for managing early childhood caries in Aboriginal preschoolers in remote communities. Although additional costs would be required for ART-HT delivery, these extra costs were less than the common willingness-to-pay benchmark of $50,000/QALY. In fact, ART-HT is highly likely to be cost-effective even when the willingness-to-pay threshold is $15,000/QALY gain, identified from the acceptability curve. While the ART-HT intervention is cost-effective, it presents an opportunity cost if resources are diverted from other programs. This necessitates a tradeoff decision regarding whether to invest in ART-HT or allocate funds to alternative health initiatives.

This is the first economic evaluation to assess the health outcomes of ART-HT in terms of the QALYs. The use of QALYs offers several advantages. First, QALYs capture both the quantity and quality of life of individuals and provide a comprehensive measure. By incorporating these 2 aspects of health, QALYs enable direct comparisons across different health conditions, interventions, and patient groups, allowing a fair and standardized evaluation of health care interventions. Second, QALYs are derived from an instrument that focuses on people’s preferences, giving a better sense of what people value in oral health rather than commonly used clinical outcome measures. Third, when QALYs are compared with the cost of care, the “value for money” provides useful information for improving efficiency in resource allocation.

Another strength of this study is the use of the ECOHIS-4D to measure the QALYs. The ECOHIS-4D converts responses from the 13 items of the ECOHIS questionnaire into utility values using algorithms and value sets specifically derived from the Australian population. This enables the accurate calculation of QALYs. While evidence demonstrating the sensitivity to change of ECOHIS-4D is forthcoming, published studies have reported that oral health–related quality-of-life measures are more precise than generic instruments are (Rogers et al. 2019; Knapp et al. 2021). Other measures are available, such as Caries Impacts and Experiences questionnaires for children (CARIES-QC) (Rogers et al., 2022) and the Dental Caries Utility Index (DCUI) (Hettiarachchi, Kularatna, et al. 2022). Both ECOHIS and CARIES-QC have been shown to be reliable and valid measures for assessing oral health–related quality of life in Australian Indigenous children (Arrow, Brennan, et al. 2021). However, the ECOHIS-4D is recommended for children younger than 7 y (Hettiarachchi, Arrow, et al. 2022), who were the participants of this study, whereas the CARIES-QC is recommended for children aged 5 to 16 y (Rogers et al. 2022).

This study is subject to several limitations. First, the 1-y time horizon constrains an assessment of long-term effects and costs associated with ART-HT and usual care. This results in a lack of comprehensive information on resource-allocation decisions over an extended period. Extrapolating costs and outcomes could help determine the potential sustained impact of ART-HT. However, as elaborated in the “Materials and Methods” section, extrapolating the results beyond the trial period would rely on assumptions that may not hold true for this specific populations, given the distinct characteristics of caries progression in Aboriginal children. With only 2 assessment time points, care should be taken when interpreting the validity and reliability of any potential extrapolated estimates, even if attempted. Further research with a longer follow-up period and more detailed data on caries progression in this population is required to better understand the long-term effects and costs of ART-HT and to inform robust policy decisions. Second, the generalizability of these findings may be limited due to the specific demographic and geographic context of the study population.

Despite the relatively modest QALYs gains observed, the culturally appropriate design of ART-HT is a significant consideration. The intervention aligns with the Cultural Safety in Healthcare for Indigenous Australians guidelines (Australian Institute of Health and Welfare 2023a), and the ethical frameworks for research with Aboriginal and Torres Strait Islander people and communities (National Health and Medical Research Council 2018), emphasizing its potential for positive outcomes within this specific context.

In summary, this trial-based cost-utility analysis provides preliminary evidence suggesting that ART-HT may offer a cost-effectiveness approach for managing early childhood caries in Indigenous preschoolers in remote communities. The results indicated potential economic advantages but should be interpreted cautiously due to the study’s limitations, particularly the short time horizon. Further research with a longer follow-up period is necessary to confirm these findings and inform robust policy decisions regarding the implementation of ART-HT in this population.

## Author Contributions

U. Tonmukayakul, contributed to conception, design, data acquisition, analysis, and interpretation, drafted and critically revised the manuscript; S. Kularatna, D. Atkinson, contributed to conception, data interpretation, drafted manuscript, critically revised the manuscript; L. Jamieson, contributed to design, data interpretation, drafted and critically revised the manuscript; P. Arrow, contributed to conception, design, data acquisition and interpretation, drafted and critically revised the manuscript. All authors gave final approval and agree to be accountable for all aspects of the work.

## Supplemental Material

sj-docx-1-jct-10.1177_23800844251346743 – Supplemental material for Cost-Utility Analysis of Treatments for Early Childhood Caries in Remote Aboriginal Communities Supplemental material, sj-docx-1-jct-10.1177_23800844251346743 for Cost-Utility Analysis of Treatments for Early Childhood Caries in Remote Aboriginal Communities by U. Tonmukayakul, S. Kularatna, D. Atkinson, L. Jamieson and P. Arrow in JDR Clinical & Translational Research
