# Supplementary material for: Cost-Utility Analysis of Treatments for Early Childhood Caries in Remote Aboriginal Communities
Source: JDR Clin Trans Res. 2025 Aug 16;11(3):368–76. doi: 10.1177/23800844251346743 (PMC13232586; doi:10.1177/23800844251346743)
Supplement: Supplementary file 1 [file sj-docx-1-jct-10.1177_23800844251346743.docx]

**Supplementary Cost-effectiveness parameter**

| **Parameters** | **Mean**  **(SE)** | **Distribution**  **(assumption)** | **Source** |
| --- | --- | --- | --- |
| ART-HT training per child | $9.25  (9.25) | Gamma distribution  (assumed mean = SE) | Project record |
| Observed ART-HT treatment fees | $439.22  (36.32) | Gamma distribution | Study records |
| Hypothetical ART-HT treatment fees | $451.52  (35.61) | Gamma distribution | Western Australia Dental Services |
| Average treatment fee in usual care | $420.60  (420.60) | Gamma distribution  (assumed mean = SE) | Study records |
| Year in the trial | 0.93  (0.1) | Normal distribution | Study records |
| Baseline ECOHIS-4D utility weights  ART-HT  Usual care | 0.91  (0.0011)  0.92  (0.0093) | Beta distribution  Beta distribution | Study records |
| Follow-up adjusted ECOHIS-4D utility weights  ART-HT  Usual care | 0.94  (0.0029)  0.94  (0.0025) | Beta distribution  Beta distribution | Study records |
| Follow-up unadjusted ECOHIS-4D utility weights  ART-HT  Usual care | 0.94  (0.0079)  0.94  (0.0089) | Beta distribution  Beta distribution | Study records |
